# Association between subclinical mastitis pathogens and passive transfer of immunity in calves

**DOI:** 10.3389/fvets.2025.1664685

**Published:** 2025-09-02

**Authors:** Gokcenur Sanioglu Golen

**Affiliations:** Department of Microbiology, Faculty of Veterinary Medicine, Aksaray University, Aksaray, Türkiye

**Keywords:** biomarker, *Escheria coli*, immunoglobulin, pathogen, *Staphylococcus aureus*

## Abstract

**Introduction:**

Colostrum plays a critical role in providing passive immunity in newborn calves, and its immunological effectiveness is closely linked to the health status of the producing cow. Subclinical mastitis, an inflammation of the mammary gland without visible clinical signs, frequently caused by pathogens such as *Staphylococcus aureus*, *Escherichia coli*, *Streptococcus agalactiae*, and *Corynebacterium bovis*, can impair colostrum quality. Haptoglobin (Hp) and serum amyloid A (SAA) are acute phase proteins that increase during infection and inflammation and may serve as biomarkers for detecting subclinical mastitis and assessing its impact on calf health.

**Methods:**

Colostrum and serum samples were collected from 20 healthy cows and 20 cows diagnosed with subclinical mastitis, along with their newborn calves. Bacteriological cultures were performed to identify mastitis pathogens. Hp, SAA, and total immunoglobulin (Ig) concentrations were measured using ELISA kits. Correlations between colostrum and calf serum biomarker levels were analyzed. Calves were monitored for diarrhea in the first week of life, and fecal samples from diarrheic calves were tested for common enteric pathogens.

**Results:**

All cows in the subclinical mastitis group were positive for at least one mastitis-causing pathogen, most frequently *S. aureus* (35%), followed by *E. coli* (30%), *S. agalactiae* (20%), and *C. bovis* (15%). Cows with subclinical mastitis had significantly higher Hp and SAA levels and lower Ig concentrations in colostrum compared with healthy cows (*p* < 0.05). Calves from the subclinical mastitis group also exhibited higher serum Hp and SAA concentrations and lower serum Ig levels (*p* < 0.05). Positive correlations were found between colostrum and calf serum Hp and SAA concentrations, while colostrum Ig showed negative correlations with both biomarkers. Four calves from the subclinical mastitis group developed diarrhea, all with low serum Ig concentrations, and fecal analysis revealed rotavirus, coronavirus, or *E. coli* K99.

**Discussion:**

Subclinical mastitis, particularly infections caused by *S. aureus* and *E. coli*, is associated with increased inflammatory markers and reduced immunoglobulin content in colostrum, leading to impaired passive immune transfer in calves. Elevated Hp and SAA in calves may indicate both passive transfer from colostrum and early activation of the neonatal immune system. These findings highlight the importance of pathogen-driven alterations in colostrum composition and support the potential of Hp and SAA as biomarkers for monitoring subclinical mastitis and predicting calf health outcomes.

## 1 Introduction

Calf immunity, development, and growth are critical factors affecting cow productivity. The first 4 week period is of critical importance in minimizing calf morbidity and mortality. However, in the neonatal period, their neonatal innate responses may not be fully developed and they are unable to form an active immune response. Neonatal calves rely primarily on passive immunity transferred from cows for protection against disease. Colostrum plays a central role in immune transfer; it contains many immune components critical for immunity such as immunoglobulins, cytokines and acute phase proteins (APPs), lipids, carbohydrates, and micronutrients, and it is also affected by subclinical mastitis pathogens (1).

In dairy farming, the health of a calf after birth is related to the quality of the colostrum it receives from its cows milk. The amount and biological activity of these components are closely related to the cow’s health status and in particular the inflammatory condition of the mammary gland (1, 2). Subclinical mastitis, a condition characterized by inflammation of the mammary tissue without obvious clinical signs, may cause alteration in colostrum content and negatively affect the transfer of passive immunity (2).

Subclinical mastitis, which is an inflammation of the mammary gland that occurs without obvious clinical symptoms, is commonly caused by pathogens such as *Staphylococcus aureus (S. aureus)*, *Escherichia coli (E. coli)*, *Streptococcus agalactiae (S. agalactiae)*, and *Corynebacterium bovis (C. bovis)*, and can negatively affect colostrum quality. Acute phase proteins like haptoglobin (Hp) and serum amyloid A (SAA) increase in response to infection and inflammation, making them potential biomarkers for identifying subclinical mastitis and evaluating its effects on calf health. Haptoglobin (Hp) and serum amyloid A (SAA) are acute phase proteins (APP) that play an important role in cattle health and can rise rapidly during infection. The increase in APP levels in milk during mastitis makes these proteins useful as markers of mammary infections or inflammations. Therefore, Hp and SAA are considered important biomarkers for both the diagnosis of infection and the early detection of subclinical mastitis (2–4). The synthesis of Hp in both the liver and mammary tissue enables the use of milk Hp concentration as an indicator of local inflammation. Recent studies have shown that Hp is not only present in systemic circulation but can also be locally produced in the mammary gland and transferred to milk (5, 6). Especially in cases of subclinical mastitis, increases in milk Hp and SAA levels may be observed, while colostrum quality and immunoglobulin (Ig) levels may decrease, which could negatively affect immune transfer to calves (7). Recent studies have reported that milk Hp levels are significantly elevated in cows with subclinical mastitis, which increases (8).

As stated by Honkala et al. (1), colostrum plays a decisive role in the immunity of newborns. Cytokines and APPs found in colostrum, especially Hp, can contribute directly to the immune development of calves beyond passive immunity. On the other hand, some studies emphasize that the M-SAA3 isoform of SAA found in colostrum has local effects, particularly in the intestinal system, and that its passage into the systemic circulation is limited. High levels of inflammatory markers such as Hp and SAA in calves with insufficient total Ig levels may indicate inadequate passive immunity transfer (9). In this context, comparing Hp, SAA, and total Ig levels measured in colostrum and calf blood may help understand the effects of subclinical mastitis on calf health.

## 2 Materials and methods

This study was conducted on a commercial dairy farm located in Aksaray, Türkiye, which maintains a large herd of lactating Jersey cows. The animals, all in their second to fourth lactation, were housed in a free-stall system under the same management and environmental conditions, milked twice daily, and clinically healthy at the time of calving. They were fed a total mixed ration formulated to meet the nutritional requirements of lactating dairy cows and had ad libitum access to clean water.

A total of 40 multiparous cows were selected from a larger population of calving animals over a two-month period. Selection criteria included the availability of complete health records and the absence of systemic illness or clinical mastitis. Within 48 h postpartum, the cows were evaluated using the California Mastitis Test (CMT), somatic cell count (SCC), and bacteriological culture of colostrum samples.

Subclinical mastitis was diagnosed based on the presence of all three of the following criteria: (1) SCC > 200,000 cells/mL, (2) a positive CMT score (≥trace), and (3) isolation of at least one mastitis-causing bacterium (*S. aureus*, *E. coli*, *S. agalactiae*, or *C. bovis*) from colostrum culture. Cows meeting these criteria were assigned to the subclinical mastitis (SCM) group (*n* = 20). The healthy control group (*n* = 20) consisted of cows with SCC < 200,000 cells/mL, negative CMT results, and no bacterial growth in culture. Group selection ensured parity and calving dates were comparable between groups.

Each calf born to a selected cow was included in the study, resulting in 40 cow–calf pairs. Colostrum samples were aseptically collected within 48 h after calving. Ten milliliters of each sample were transferred into sterile tubes and stored for microbiological analysis. Cultures were performed on 5% sheep blood agar and MacConkey agar, and incubated aerobically at 37 °C for 24 h. Bacterial colonies were identified using standard microbiological techniques, including morphology, Gram staining, and biochemical testing.

Blood samples were obtained from the jugular vein of each calf between 24 and 36 h of life using sterile vacutainer tubes. Calves were fed colostrum within the first 2 h after birth and again within the first 12 h, receiving a total of approximately 4–6 liters during the first day of life.

Colostrum samples were centrifuged at 3,000 rpm for 15 min to separate the supernatant. Calf serum samples were stored at −20 °C until analysis.

The sample size (*n* = 20 per group) was determined based on previous studies examining colostrum quality and passive immunity in calves, with the goal of detecting significant differences in immunoglobulin and acute phase protein levels. The design achieved 80% statistical power at a significance level of 0.05, while also accounting for practical constraints such as the availability of cows meeting the inclusion criteria during the calving period.

Hp concentrations in colostrum and calf serum were measured using a commercial ELISA kit [Bioassay Technology Laboratory, Cat. No: E1035Bo; (10)]. SAA levels were determined using an ELISA kit from MyBioSource (Cat. No: MBS036405). Total immunoglobulin (Ig) concentrations in calf serum were assessed using an ELISA kit from Elabscience (Cat. No: BOFI00152). Immunoglobulin concentrations in colostrum were directly measured using a commercial bovine Ig ELISA kit (Elabscience, Cat. No: BOFI00152) according to the manufacturer’s instructions. Colostrum samples were diluted 1:10 prior to analysis. Optical density was read at 450 nm using a microplate reader, and concentrations were calculated from a standard curve. In addition to calf serum Ig measurements, these colostrum Ig values were used to assess colostrum quality and their correlation with passive transfer efficiency. Sample dilutions were prepared according to the manufacturer’s instructions: 1:10 for colostrum and 1:1000 for serum. Optical density was read at 450 nm using a microplate reader, and concentrations were calculated based on standard curves.

All statistical analyses were performed using IBM SPSS Statistics version 26.0. Data distribution was assessed using the Shapiro–Wilk test to determine the appropriate statistical method. Normally distributed variables were analyzed using independent samples *t*-tests, while Pearson correlation analysis was used to assess relationships between continuous variables. Statistical significance was defined as *p* < 0.05. Bonferroni correction was applied for multiple comparisons.

Fresh fecal samples were collected from calves exhibiting diarrhea within the first week of life. These samples were analyzed for the presence of rotavirus, coronavirus, and enterotoxigenic *E. coli* (K99 antigen) using the IDEXX Rota-Corona-K99 ELISA test kit, following the manufacturer’s instructions. The results were analyzed using the same statistical parameters as described above.

## 3 Results

Bacteriological analysis of colostrum samples obtained from cows in the subclinical mastitis (SCM) group showed that all 20 milk samples (100%) were positive for at least one mastitis-causing bacterial pathogen. The most frequently isolated microorganism was *S. aureus*, detected in 35% of the samples, followed by *E. coli* (30%), *S. agalactiae* (20%), and *C. bovis* (15%), as shown in Table 1. No bacterial growth was identified in any of the samples from the healthy control group.

**Table 1:** Bacterial pathogens isolated from milk samples in the subclinical mastitis group.

Pathogen	Number of isolated samples (*n* = 20)	Percentage (%)
*S. aureus*	7	35%
*E. coli*	6	30%
*S. agalactiae*	4	20%
*C. bovis*	3	15%
Total	20	100%

Analysis of inflammatory markers revealed significant differences between groups. Hp and SAA levels in the milk of cows diagnosed with subclinical mastitis were significantly higher than those in the healthy group. Specifically, the mean milk Hp concentration in the SCM group was 85.3 ± 12.5 μg/mL, compared to 49.6 ± 10.8 μg/mL in the healthy group (*p* = 0.004). Similarly, milk SAA concentrations were 63.8 ± 9.7 μg/mL in the SCM group and 35.2 ± 7.3 μg/mL in the control group (*p* = 0.012). In the corresponding calves, serum Hp and SAA levels were also significantly elevated in the SCM group. Calf serum Hp levels averaged 93.1 ± 11.2 μg/mL in the SCM group and 60.7 ± 9.4 μg/mL in the healthy group (*p* = 0.001), while SAA levels were 71.4 ± 10.1 μg/mL and 41.5 ± 8.2 μg/mL, respectively (*p* = 0.008).

In terms of immune status, immunoglobulin (Ig) concentrations in colostrum were significantly lower in the SCM group (mean ± SD: 45.8 ± 7.6 mg/mL) compared to the healthy group (63.4 ± 8.2 mg/mL; *p* = 0.002). Similarly, total Ig concentrations in calf serum were significantly lower in the SCM group (19.7 ± 3.8 mg/mL) compared to the healthy group (27.6 ± 4.2 mg/mL; *p* = 0.023). A strong positive correlation was found between colostrum Ig and calf serum Ig levels (*r* = 0.71, *p* < 0.05). Colostrum Ig also showed moderate negative correlations with colostrum Hp (*r* = −0.52, *p* < 0.05) and SAA (*r* = −0.49, *p* < 0.05), indicating that mammary inflammation may be associated with reduced immunoglobulin content.

**Figure 1:**
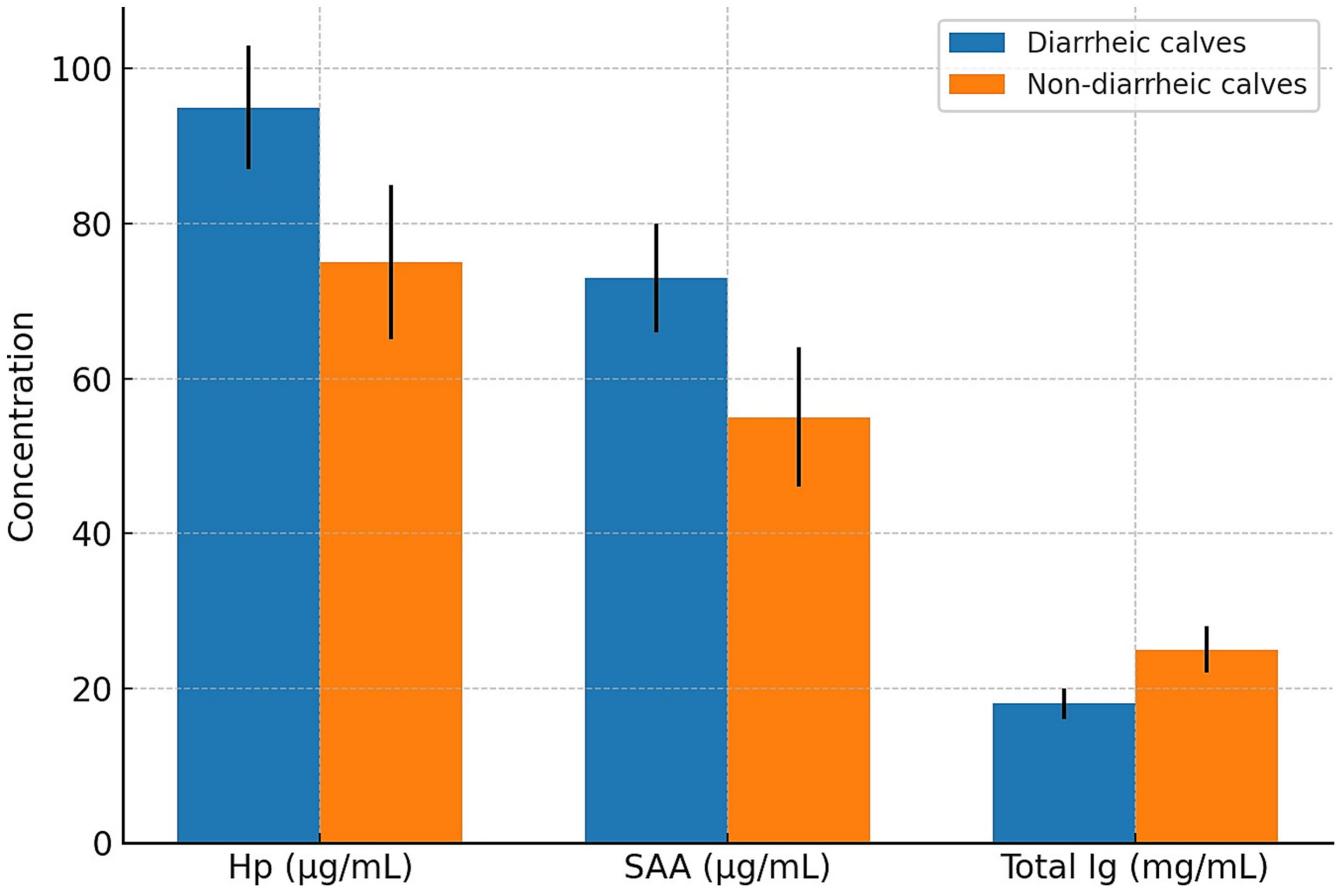
Comparison of haptoglobin (Hp), serum amyloid A (SAA), and total immunoglobulin (Ig) concentrations between diarrheic (*n* = 4) and non-diarrheic (*n* = 36) calves. Bars represent mean ± standard deviation (SD). All diarrheic calves were born to cows with subclinical mastitis.

During the first week of life, diarrhea was observed in four of the forty calves (10%) included in the study. All affected calves were from the subclinical mastitis group, representing an incidence of 20% within that group, while no cases of diarrhea were reported in the healthy control group. Although this difference was not statistically significant (Fisher’s exact test, *p* = 0.10), the finding may be biologically relevant. Each of the diarrheic calves had serum immunoglobulin levels below 20 mg/mL, consistent with failure of passive transfer. Pathogen screening via IDEXX Rota-Corona-K99 ELISA tests identified rotavirus in two calves, coronavirus in one calf, and K99-positive *E. coli* in one calf. These findings suggest a possible link between maternal subclinical mastitis, impaired passive immunity, and increased neonatal susceptibility to enteric pathogens (Figure 1).

## 4 Discussion

This study aimed to investigate the relationship between Hp and SAA levels in the colostrum of cows with subclinical mastitis (SCM) and systemic concentrations of Hp, SAA, and total immunoglobulin (Ig) in their neonatal calves. The findings demonstrated that both Hp and SAA levels were significantly elevated in the colostrum and serum of the SCM group, whereas total Ig concentrations in the calves were significantly lower compared to the healthy group. These results suggest that subclinical mastitis affects colostrum composition and may impair passive immune transfer in calves (Table 2).

**Table 2:** Mean ± standard deviation (SD) concentrations of haptoglobin (Hp), serum amyloid A (SAA), and total immunoglobulin (Ig) in colostrum and calf serum according to maternal health status.

Parameter	Milk (subclinical)	Milk (healthy)	Calf serum (subclinical)	Calf serum (healthy)	*p*-value
Haptoglobin (μg/mL)	85.3 ± 12.5	49.6 ± 10.8	93.1 ± 11.2	60.7 ± 9.4	0.004
SAA (μg/mL)	63.8 ± 9.7	35.2 ± 7.3	71.4 ± 10.1	41.5 ± 8.2	0.012
Total Ig (mg/mL)	45.8 ± 7.6	63.4 ± 8.2	19.7 ± 3.8	27.6 ± 4.2	0.002* (milk)/0.023 (serum)

Values are compared between cows diagnosed with subclinical mastitis and healthy controls. Ig levels were assessed in both colostrum and calf serum. *p*-values are shown for between-group comparisons.

Mastitis is characterized by local inflammation and increased permeability of the mammary epithelium, which alters the immunological profile of colostrum. Acute phase proteins (APPs), including Hp and SAA, are known to increase in colostrum during inflammatory responses. Previous studies have identified Hp as a promising diagnostic biomarker in subclinical mastitis. Carinelli et al. (2) demonstrated its diagnostic utility, and in a subsequent study, the same group developed a biosensor-based method for rapid, field-based Hp detection in milk. Carinelli et al. (2) also showed that milk Hp levels were both sensitive and specific for distinguishing subclinical from clinical mastitis, and that Hp responses varied by pathogen.

In our study, *S. aureus* and *E. coli* were identified as the predominant pathogens in subclinical mastitis (SCM) milk samples, a finding that is in line with the well-established role of these bacteria as major causative agents of intramammary infections in dairy cows. The frequent isolation of these pathogens not only reflects their widespread prevalence in bovine mastitis cases but also underscores their significant impact on udder health and milk quality. Importantly, the association of these microorganisms with acute phase responses highlights the intricate interplay between pathogen type and host immune activation.

Specifically, the markedly elevated levels of haptoglobin (Hp) observed in samples positive for *S. aureus* infection are consistent with previous reports (11), suggesting that this pathogen induces a more pronounced acute phase response compared to other bacteria. This may be attributable to the chronic and often persistent nature of *S. aureus* infections, which are known to trigger sustained inflammatory signaling and tissue damage. In contrast, while E. coli is also capable of stimulating acute phase protein synthesis, its effects may be more transient due to the typically acute but shorter duration of coliform infections.

Our findings also align with the results of Åkerstedt et al. (12), who assessed Hp and SAA in the milk of clinically healthy cows and reported a correlation between elevated APPs and changes in milk composition, including protein and lactose levels. In their multivariate analysis, high somatic cell counts were associated with increased APP concentrations and reduced casein and lactose content. These changes may negatively affect both milk processing characteristics and the ability of colostrum to support passive immunity in calves.

The synthesis of Hp locally in the mammary gland, in addition to hepatic production, supports the utility of colostrum as a matrix for evaluating local inflammation (4). However, differences between serum and plasma Hp measurements have been noted (5), and should be considered when comparing biological fluids. Despite this, we observed a moderate positive correlation between Hp levels in colostrum and those in calf serum, supporting a potential immunological link between maternal colostrum and neonatal systemic responses.

The elevated Hp and SAA levels in calf serum suggest activation of the neonatal inflammatory response. Whether these proteins are transferred passively or reflect endogenous synthesis is a matter of debate. Honkala et al. (1) proposed that some APPs can cross the intestinal barrier shortly after birth, contributing to early immune modulation. In particular, IL-6-mediated stimulation of Hp synthesis may shift the neonatal immune response from an innate Th2-dominant profile toward a more adaptive Th1-dominant state. Hendriks et al. (8) also demonstrated that milk Hp concentrations are highest within the first 24 h postpartum, consistent with our sampling timeline and findings.

In contrast, the extent to which SAA is absorbed systemically remains uncertain. Honkala et al. (1) noted that SAA isoforms in colostrum primarily exert localized effects on the gastrointestinal tract and are minimally present in systemic circulation. This could explain the weaker correlation between colostral and serum SAA levels in our study. Orro et al. (13) also observed that systemic SAA levels in calves increase in response to microbial colonization and vary with age, suggesting that early postnatal inflammation may reflect environmental exposures rather than passive SAA transfer.

In this study, colostrum Ig concentrations were directly measured and found to be significantly reduced in the SCM group compared to healthy cows, confirming that subclinical mastitis compromises colostrum quality. Total Ig concentrations are a well-established indicator of passive transfer effectiveness. The significantly lower Ig levels observed both in colostrum and in the serum of calves from the SCM group provide direct evidence that subclinical mastitis compromises colostrum quality. The strong positive correlation between colostrum Ig and calf serum Ig levels in our study aligns with previous findings by Furman-Fratczak et al. (9) and Chigerwe et al. (14), who reported that colostrum Ig concentration is the primary determinant of passive transfer success. The observed negative correlation between colostrum Ig and colostral Hp/SAA concentrations supports the concept that inflammation in the mammary gland can impair immunoglobulin synthesis, secretion, or stability, as suggested by Baumrucker and Bruckmaier (15). This relationship underscores the importance of controlling subclinical mastitis not only to reduce local inflammation but also to preserve the immunological value of colostrum. According to Furman-Fratczak et al. (9), serum IgG concentrations below 10 g/L are indicative of failure of passive transfer (FPT), which increases susceptibility to infectious diseases and mortality in neonatal calves. In our study, the negative correlation between total Ig and APPs (Hp and SAA) suggests that inflammatory colostrum may impair immunoglobulin absorption or reflect concurrent infection risks in the calf.

Džermeikaitė et al. (16) reported that high SAA levels in milk are associated with low lactose concentrations, implicating mammary epithelial dysfunction in subclinical inflammation. This supports the idea that inflammation-induced alterations in colostrum composition may impact neonatal health. A limitation of this study is that potential confounding factors such as parity and environmental conditions, although standardized in our herd, could still influence colostrum composition and calf immunity.

The occurrence of diarrhea in four calves from the SCM group, all of which had serum Ig levels below 20 mg/mL, further highlights the clinical implications of impaired passive immunity. The identification of enteric pathogens—rotavirus, coronavirus, and K99 + *E. coli*—in these calves underscores their increased vulnerability to infections. Although the sample size limits statistical significance (Fisher’s exact test, *p* = 0.10), the findings are consistent with prior research linking FPT with higher morbidity. However, the small number of diarrheic calves (*n* = 4) limits the statistical power of the subgroup analysis. Although Fisher’s exact test was applied, the small sample size means these results should be interpreted with caution, and larger-scale studies are needed before drawing firm causal conclusions. Honkala et al. (1) emphasized the role of colostrum-derived APPs in shaping neonatal immune development, while Furman-Fratczak et al. (9) stressed the importance of achieving adequate IgG levels to reduce disease incidence.

Our findings also corroborate the study by Kabu et al. (17), which reported significantly elevated Hp and SAA levels in diarrheic calves compared to healthy controls. Similarly, Hajimohammadi et al. (18) found that APP concentrations rose in parallel with diarrhea severity and were correlated with clinical signs, suggesting their potential as diagnostic and prognostic biomarkers in neonatal disease. Şeliman et al. (19) reported significantly elevated Hp and SAA levels in cattle with dermatophytosis before treatment, which decreased following therapy. Similarly, increased levels of Hp and SAA in the colostrum of cows with subclinical mastitis were mirrored in their calves, along with reduced passive immunity. Both studies highlight that Hp and SAA are reliable biomarkers of infection and useful indicators for monitoring disease progression and treatment response.

## 5 Conclusion

In conclusion, this study demonstrated that subclinical mastitis, most frequently associated with *S. aureus* and *E.coli*, increases haptoglobin (Hp) and serum amyloid A (SAA) concentrations in colostrum while reducing its immunoglobulin (Ig) content. Calves born to SCM-affected cows exhibited elevated serum Hp and SAA, reduced Ig levels, and a higher incidence of diarrhea, indicating impaired passive transfer of immunity and increased susceptibility to enteric pathogens. These findings highlight the pathogen-driven alterations in colostrum composition as a critical factor influencing neonatal health. Early detection and control of SCM are therefore essential not only to protect colostrum immunological quality but also to prevent passive transfer failure and reduce the risk of calf diarrhea.

## Data Availability

The original contributions presented in the study are included in the article/supplementary material, further inquiries can be directed to the corresponding author.
